# X-linked Lymphoproliferative Disease Type 1 Presenting as Lymphoma in a Male Patient With Atypical Common Variable Immunodeficiency Features: A Case of Delayed Diagnosis

**DOI:** 10.7759/cureus.86800

**Published:** 2025-06-26

**Authors:** Tuqa A Abdulsalam, Sura Ahmed

**Affiliations:** 1 General Pediatrics, Al Jalila Children's Speciality Hospital, Dubai, ARE; 2 Pediatrics, Al Jalila Children's Speciality Hospital, Dubai, ARE

**Keywords:** burkitt lymphoma, cvid mimic, ebv-negative lymphoma, hematopoietic stem cell transplantation, pediatric immunology, primary immunodeficiency, sh2d1a mutation, x-linked lymphoproliferative disease

## Abstract

X-linked lymphoproliferative disease type 1 (XLP1) is a rare, often fatal primary immunodeficiency (PID) caused by mutations in the SH2D1A gene that result in an uncontrolled immune response to Epstein-Barr virus (EBV) infection. However, a small number of patients do not encounter EBV exposure and exhibit clinical and immunological features similar to those of common variable immunodeficiency (CVID), leading to delayed diagnosis. Herein, we report the case of a 13-year-old boy diagnosed at eight years of age with CVID after he experienced recurrent pneumonias, hypogammaglobulinemia, and chronic bronchiectasis. He was given monthly intravenous immunoglobulin (IVIG) for five years. At 13 years of age, he presented with abdominal pain, and imaging studies demonstrated a bulky retroperitoneal mass, which was confirmed by biopsy to be Burkitt lymphoma. Immunophenotyping revealed low CD4⁺ T cells and B cells, as well as an inverted CD4⁺ to CD8⁺ T-lymphocyte ratio (CD4⁺/CD8⁺). A diagnosis of XLP1 was established through whole-exome sequencing, in the presence of negative EBV serology, which revealed a hemizygous pathogenic variant of SH2D1A. He was managed with chemotherapy and evaluated for hematopoietic stem cell transplant. This case highlights the significance of including monogenic immunodeficiencies in the differential diagnosis of male patients with atypical CVID phenotypes and early-onset cancer. Presymptomatic genetic diagnosis can enable early and potentially curative diagnosis and treatment.

## Introduction

X-linked lymphoproliferative disease (XLP) is a rare and life-threatening primary immunodeficiency (PID) characterized by impaired lymphocyte activation and cytotoxic activity due to mutations. It is inherited in an X-linked manner and is confined almost exclusively to male patients; the estimated first occurrence is fewer than one in 1,000,000 live births [1]. Two clinical subtypes have been established: XLP type 1 (XLP1), caused by mutations in the SH2D1A gene, which encodes the signaling lymphocytic activation molecule (SLAM)-associated protein (SAP), and XLP type 2 (XLP2), associated with mutations in the XIAP gene, which encodes the X-linked inhibitor of apoptosis protein [2]. These mutations prevent T-cell and natural killer (NK)-cell cytotoxicity, disrupting the immune synapse and leading to uncontrolled lymphocyte activation, especially in the presence of Epstein-Barr virus (EBV) [1-3]. A report by Purtilo et al. in 1975 described familial fatal mononucleosis following EBV infection and was the first to demonstrate the X-linked inheritance pattern of this syndrome [2].

The clinical manifestations of XLP1 are heterogeneous, ranging from presentation with fulminant infectious mononucleosis, hemophagocytic lymphohistiocytosis (HLH), and/or B-cell lymphomas (notably Burkitt lymphoma) to autoimmune cytopenias and progressive hypogammaglobulinemia [2-4]. Despite EBV being a major initiating factor in most patients, a subset of male patients have EBV-independent disease, with persistent hypogammaglobulinemia, recurrent infections, and bronchiectasis, suggestive of common variable immunodeficiency (CVID) [5,6]. Such clinical similarities can lead to delayed or incorrect diagnoses, particularly in patients lacking a family history or EBV exposure. The pathogenic variant of SH2D1A results in SAP deficiency, which disrupts the SLAM receptor pathway, causing ineffective T-cell-mediated clearance of EBV-infected B cells and dysregulated cytokine production [1,3]. Genetic testing is crucial in atypical cases, as it enables diagnosis and allows prompt referral for hematopoietic stem cell transplantation (HSCT), which remains the only curative treatment. Early detection is vital since HSCT prospects are better when performed before the onset of EBV infection or malignancy [6,7].

## Case presentation

**Figure 1:**
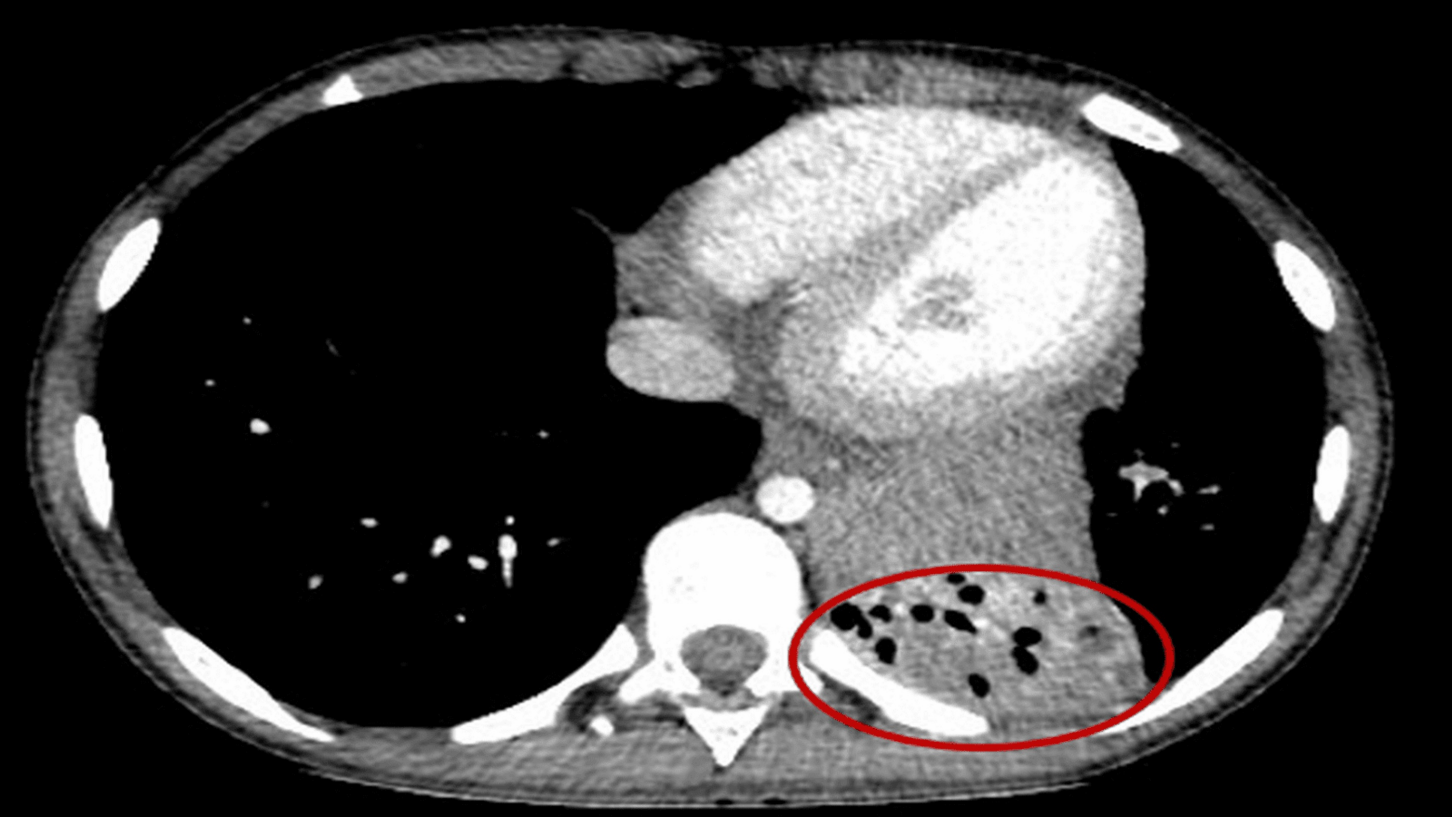
High-resolution chest CT (axial view) showing left lower lobe collapse with cystic bronchiectasis and calcified hilar lymphadenopathy. CT: computed tomography

A previously healthy male child began experiencing recurrent respiratory infections around the age of four. These episodes necessitated several admissions due to bilateral lobar pneumonia, requiring intravenous antibiotics. In the next several years, he developed a productive cough, shortness of breath on exertion, and wheezing, which were initially treated with bronchodilators and inhaled steroids. At seven years of age, there were more frequent and severe lower respiratory tract infections, necessitating investigations. A high-resolution computed tomography (CT) of the chest showed chronic collapse of the left lower lobe, cylindrical and cystic form of bronchiectasis, and calcified lymphadenopathy in the region of the left hilum. A separate soft tissue mass was also seen at the medial aspect of the collapsed lung, indicating mediastinal extension (Figure 1).

Sweat chloride and cystic fibrosis gene analyses were negative. Laboratory investigation demonstrated persistent hypogammaglobulinemia with severely decreased IgG (250 mg/dL) and IgA (30 mg/dL) and increased IgM (380 mg/dL).

Following these immunological studies, a diagnosis of CVID was made, and he was treated with monthly intravenous immunoglobulin (IVIG) infusions and azithromycin as a prophylaxis. Lymphocyte subset analysis on initial presentation showed a decreased number of CD4⁺ T cells, a decreased number of CD19⁺ B cells, and an inverted CD4⁺ to CD8⁺ T-lymphocyte ratio (CD4⁺/CD8⁺), all of which were indicative of underlying immune dysregulation. Despite repeated IVIG infusions, he presented with multiple infections during the first year of follow-up, including hospital admissions for pneumococcal pneumonia and several episodes of lower respiratory infections requiring intravenous antibiotics. On sequential chest images, bronchiectasis and left lower lobe collapse were noted to be persistent. From the ages of nine to 12 years, his respiratory symptoms remained stable, attributed to his regular airway clearance therapy and maintenance of IVIG infusions. However, at 13 years of age, during an elective infusion clinic visit, he complained of one month of non-radiating epigastric abdominal pain, not associated with meals, and it resolved spontaneously. He denied constitutional symptoms, including weight loss, night sweats, or fever, and had a chronic cough.

On physical examination, the child was pale with vital signs within range. Chest examination revealed bilaterally consistent basilar crepitations. Abdominal examination revealed a non-tender hard mass of approximately 10 cm in diameter in the right iliac region and splenomegaly 4 cm below the costal margin. Of note, an abdominal ultrasound performed one month before admission was normal, contributing to the diagnosis of a rapidly growing mass. On repeat testing, a normal white blood cell (WBC) count (7.2 × 10^9^/L), hemoglobin (12.4 g/dL), and platelet count (280 × 10^9^/L) were observed using a complete blood count investigation, as outlined in Table 1.

**Table 1:** Complete blood count WBC: white blood cell

Parameter	Result	Reference Range
WBC count	7.2 × 10⁹/L	4.5 - 13.5 × 10⁹/L
Hemoglobin	12.4 g/dL	11.5 - 15.5 g/dL
Platelet count	280 × 10⁹/L	150 - 400 × 10⁹/L

**Figure 2:**
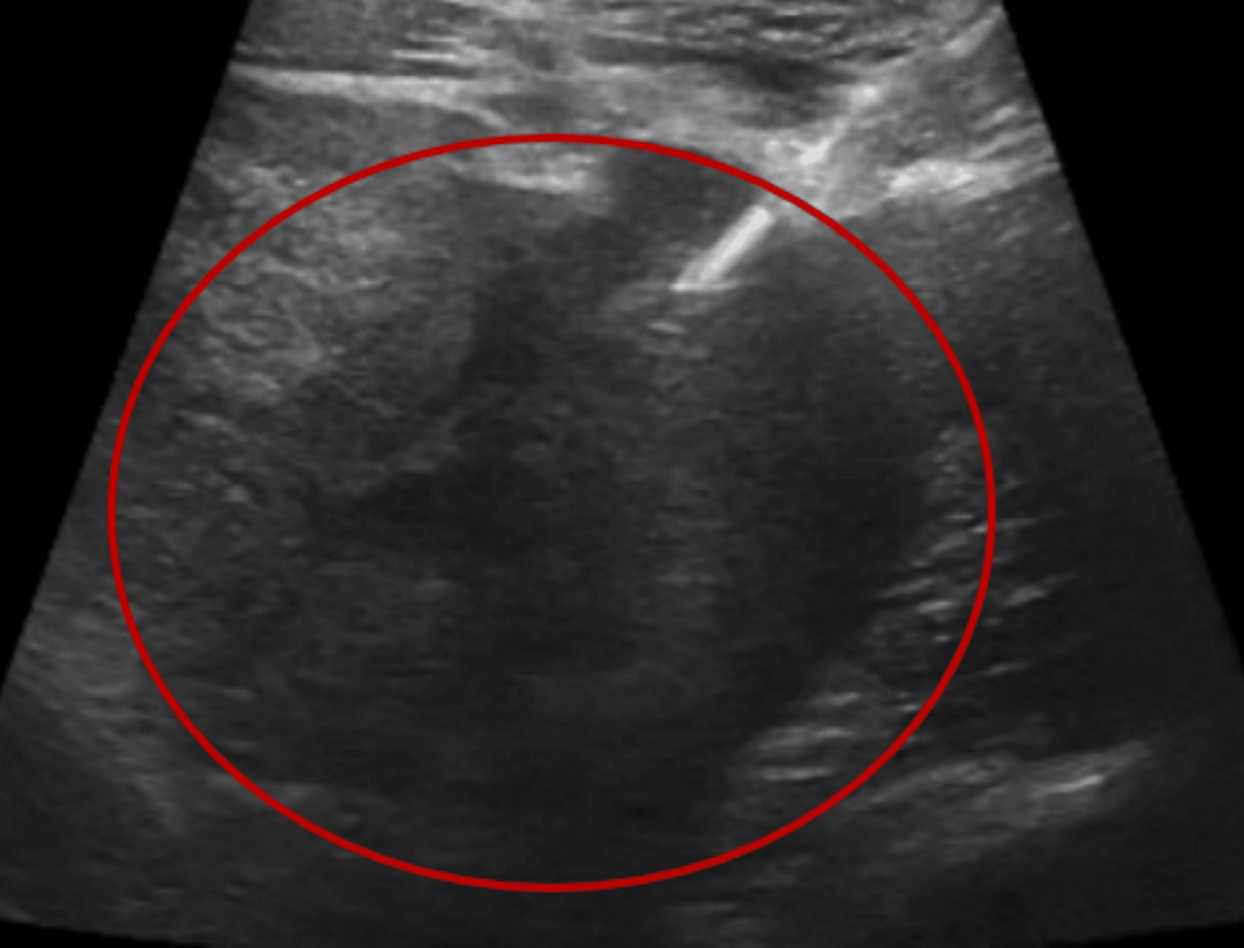
Abdominal ultrasonography revealing a large retroperitoneal mass.

Abdominal ultrasonography revealed a large heterogeneous mass in the right retroperitoneum, size 17 × 8 cm, with an anterior and medial deviation of the right kidney (Figure 2).

**Figure 3:**
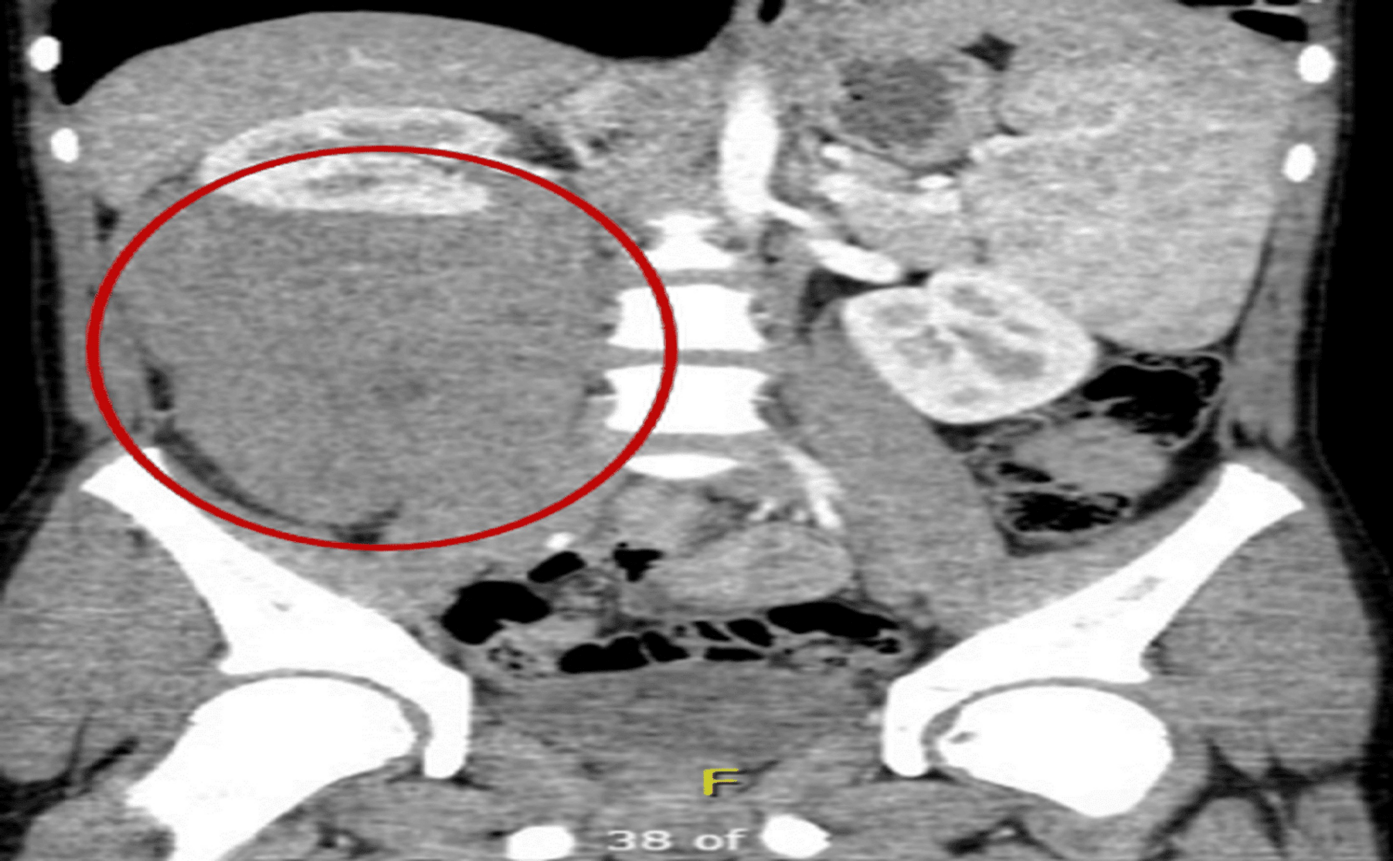
Contrast-enhanced abdominal and pelvic CT confirming a right perinephric mass. CT: computed tomography

Enhanced CT of the abdomen and pelvis demonstrated a right perinephric soft tissue mass with scalloping of the renal margins, close to the inferior vena cava and right common iliac vessels (Figure 3).

An ultrasound-guided core biopsy was performed, which on histological examination showed a high-grade malignancy of round blue cells consistent with a B-cell lymphoma. Bone marrow aspirate demonstrated mild hypocellularity, preserved trilineage hematopoiesis, and no evidence of malignant infiltration.

Due to the young age of the patient's onset of lymphoma, history of frequent infections, and immunoglobulin abnormalities, a more detailed immunologic and genetic workup was undertaken. Flow cytometry found diminished CD4⁺ T cells (25%) and CD19⁺ B cells (6%), preserved NK cells (5%), and an inverted CD4⁺/CD8⁺ ratio of 0.8.

Whole-exome sequencing revealed a hemizygous mutation in the SH2D1A gene, which confirmed the diagnosis of XLP1. EBV IgG and IgM antibodies were also negative. However, EBV polymerase chain reaction (PCR) of peripheral blood and EBV-encoded RNA test of lymph node tissue were not performed, which made it difficult to rule out viral involvement definitively.

The patient was referred to a dedicated pediatric oncology center, and he was treated with systemic chemotherapy following pediatric B-cell non-Hodgkin lymphoma schedules. The patient was scheduled to undergo allogeneic HSCT, but it was postponed because structurally damaged lung segments of bronchiectasis would increase the risk of perioperative management. At the last follow-up, the patient was in clinical remission with strict multidisciplinary surveillance.

## Discussion

XLP1 is a rare and devastating PID caused by mutations in the gene SH2D1A, which encodes SAP [1]. The function of cytotoxic lymphocytes, particularly in controlling CD8⁺ T-cell and NK-cell-mediated killing of EBV-infected B cells, depends on SAP [3]. In the absence of SAP, the inability to clear EBV-infected cells can lead to a hyperactive immune response, cytokine storm, and lymphoproliferative diseases, such as HLH and lymphomas. Nevertheless, the full spectrum of XLP1 is broader than EBV disease and encompasses antibody deficiency, recurrent infections, and B-cell tumors, also in EBV-naive subjects [4].

This case highlights the subversive CVID-like phenotype associated with XLP1. For several years, the patient suffered from repeated lower respiratory tract infections, progressive bronchiectasis, and persistent hypogammaglobulinemia - features compatible with CVID. CVID is a diagnosis of exclusion, and it is now becoming increasingly evident that it consists of genetically defined subgroups. In a multicenter cohort, nearly 10% of patients diagnosed with CVID were ultimately reclassified as having XLP1 following confirmation of genetic testing, mainly in male patients presenting with early-onset disease, elevated IgM levels, and lymphoproliferative complications [4].

The diagnosis was initially overlooked in the present patient since the early evidence of immunosuppression was subtle, and it was not accompanied by symptoms suggestive of disease related to EBV. Notably, this patient exhibited persistently high IgM levels, low IgG and IgA levels, and a reversed CD4⁺/CD8⁺ ratio, indicating a failure of SAP. However, these were at first erroneously attributed to polygenic CVID. A significant turning point in the course of the disease was marked by the spontaneous emergence of an enlarging abdominal mass, which was later diagnosed as Burkitt lymphoma. This oncologic emergency is commonly observed among patients with XLP1. Of note, the ultrasound of the abdomen just one month previously had been entirely normal, highlighting the highly aggressive behavior of the tumor and the need for rapid reassessment of the initial diagnosis. This clinical crossroad illustrates a significant diagnostic dilemma in a young male patient with CVID who presented with an onset of lymphoma or HLH. A monogenic cause, such as XLP1, should be reconsidered early on [1,7].

Of note, EBV serology was negative, confirming that XLP1 may occur in the absence of a viral trigger. Classic XLP1 is characterized by a fulminant EBV infection; however, 30-40% of patients, particularly young children, may develop the disease with no evidence of EBV. Moreover, the cause of EBV-negative lymphoma in XLP1 is still not well described, partly due to defects in SLAM family signaling and disorganized B-cell expansion [3,5].

The immunologic phenotype of this patient was consistent with a diagnosis of XLP1: CD4⁺ T-cell reduction, decreased B cells, and increased NK cells, which are commonly found in SAP deficiency [1]. However, the lymphocyte subsets did not confirm the diagnosis because of overlapping abnormalities with other immunodeficiency syndromes. Whole-exome sequencing confirmed the hemizygous pathogenic variant of SH2D1A, diagnosing XLP1. This is consistent with the proliferation of calls for early consideration of genomic testing in patients manifesting unexplained hypogammaglobulinemia, elevated IgM, or distinct CVID phenotypes, particularly with coexisting lymphoproliferation or cytopenias [5].

From a therapeutic standpoint, HSCT is the only curative treatment for XLP1 [3]. Results are best when HSCT is performed electively before exposure to EBV and emergence of malignancy. In historical analysis, five-year survival was >80% in patients who underwent preventive transplantation compared to <40% in those transplanted from HLH or lymphoma [4,7]. While this patient presented post-lymphoma, early remission and referral for transplant continue to represent excellent prognostic features.

This case contributes to the growing body of clinical knowledge about EBV-negative XLP1 mimicking CVID and reinforces several key principles. Firstly, clinicians should consider the diagnosis of XLP1 in young male patients with hypogammaglobulinemia and non-infectious complications (e.g., malignancy and HLH). Secondly, elevated IgM levels should favor early SAP deficiency in the differential diagnosis. Third, genetic testing should not be delayed, even for suspected classic CVID. And lastly, the diagnosis of XLP1 has significant consequences - not only for a patient's prognosis, but also for counseling, family planning, and transplant strategy.

## Conclusions

This case underscores the diagnostic dilemmas in male patients with XLP1 who present without EBV-associated illness but with features mimicking CVID. In children with recurrent infections, bronchiectasis, and persistently elevated IgM, a diagnosis of B-cell lymphoma should prompt early re-evaluation for a monogenic immune defect. Genetic testing plays a pivotal role in redefining the diagnosis and guiding curative treatment. Early identification of atypical immunodeficiency patterns, such as hypogammaglobulinemia with lymphoproliferative features, is essential to prevent missed diagnoses and to facilitate timely intervention. This case serves to illustrate the expanding role of genomic medicine in challenging conventional classifications of immunodeficiency and the importance of maintaining a high level of clinical suspicion for primary immunodeficiency syndromes. 
